# Anti-human-cytomegalovirus immunoglobulin G levels in glioma risk and prognosis

**DOI:** 10.1002/cam4.44

**Published:** 2013-02-03

**Authors:** E. Susan Amirian, Deborah Marquez-Do, Melissa L. Bondy, Michael E. Scheurer

**Affiliations:** 1 Dan L. Duncan Cancer Center, Baylor College of MedicineHouston, Texas; 2 Department of Pediatrics, Baylor College of MedicineHouston, Texas

**Keywords:** Brain neoplasms, glioma, human cytomegalovirus, immunoglobulin G, immunoglobulin M, risk factors

## Abstract

The role of human cytomegalovirus (HCMV) in glioma development and progression remains controversial. The purpose of our study was to assess the potential associations between anti-HCMV antibodies (immunoglobulin G [IgG] and immunoglobulin M [IgM]) and glioma risk and prognosis using data from the Harris County Case–Control Study. Multivariable logistic regression models were utilized to estimate odds ratios and 95% confidence intervals (CI) for the associations between glioma status and antibody levels among glioma cases (*n* = 362) and cancer-free controls (*n* = 462). Hazard ratios and 95% CIs were calculated using Cox proportional hazards regression, adjusting for age, race, and sex, to determine if antibody levels were associated with survival over time among cases. Among IgG-positive participants, increasing anti-HCMV IgG levels were associated with decreasing glioma risk (*P* for trend = 0.0008), and those with the lowest level of anti-HCMV IgG (<10 U/mL) had the highest glioma risk, controlling for age, sex, and race/ethnicity (OR: 2.51, 95% CI: 1.42–4.43). Antibody levels were not associated with survival among glioma cases. Our study contributes new evidence toward the potential importance of the direct and indirect effects of HCMV infection in gliomagenesis.

## Introduction

In the United States, the incidence of malignant glioma is less than 7 per 100,000 person-years, with a lifetime risk of less than 0.7% [1,2], and yet this rare disease accounts for a disproportionate amount of cancer deaths, an estimated 4% [3]. Median survival time for glioblastoma patients is slightly over a year, and there have been few therapeutic advances over time for this highly fatal cancer. Identification of glioma risk factors has also proven to be extremely challenging, despite numerous studies on environmental exposures and genetic polymorphisms [1, 4, 5]. However, a consistent trend observed in the recent literature is that immunomodulatory factors may predict glioma risk [6–10].

Since the 1950s, studies have reported associations between immune-related conditions and brain and other cancers [8, 11–14]. For glioma specifically, the last two decades have yielded multiple studies showing associations between glioma risk and various immunological indicators, such as a history of asthma and allergies [10, 15, 16], the use of antihistamines or nonsteroidal anti-inflammatory drugs (NSAIDs) [7,17], immunoglobulin E (IgE) levels [16, 18, 19], antivaricella-zoster virus immunoglobulin G (IgG) levels [12,20], and functional single nucleotide polymorphisms (SNPs) in inflammation-related genes [19, 21, 22]. Cumulatively, the majority of these studies have indicated that, in general, immunological factors that represent heightened levels of inflammation or anticancer immunosurveillance seem to be associated with lower glioma risk, whereas factors that may have immunosuppressive effects (i.e., regular, long-term use of antihistamines) tend to be associated with higher glioma risk.

Although findings on the role of viruses in glioma susceptibility have been less consistent [23,24], a few potential key players have been identified, such as two common herpesviruses, human cytomegalovirus (HCMV) and varicella-zoster virus (VZV). In fact, based on currently available evidence, it appears that HCMV DNA sequences and viral gene products are present in a majority of malignant gliomas [3,24], and some have hypothesized that HCMV is capable of establishing a type of persistent infection that does not fit with standard definitions of lytic or latent infections [24]. This persistent infection, in which certain viral genes are expressed without contributing to a significant amount of host cell lysis, may alter pro- and anti-inflammatory cytokine production levels in the host. HCMV is capable of immune evasion and modulation, and is considered an oncomodulatory, rather than oncogenic, virus because of a lack of oncoprotein expression. Thus, it follows that the impact of HCMV on glioma risk and prognosis could potentially be more related to viral manipulation of immunological factors and the host's immune response toward the virus, rather than virus-related host cell damage or a direct carcinogenic effect of a viral gene product.

The possible involvement of HCMV in glioma development is of particular interest, given both the consistently observed associations between glioma risk and immunomodulatory factors [8] and the extremely high prevalence of this immunoevasive virus in malignant glioma tissues. Thus, in this study, we sought to assess the potential associations between anti-HCMV antibody levels and glioma risk and prognosis, using data from the Harris County Case–Control Study (HCCCS).

## Materials and Methods

### Study population and data collection

Detailed information on the HCCCS has been provided elsewhere [7]. Briefly, cases consisted of adults over the age of 18 years with newly diagnosed, histologically confirmed glioma (ICD-O-3 codes 9380–9481), identified between 2001 and 2006 by hospital physicians in and around Harris County, Texas. Pathology specimens were reviewed by the study neuropathologist to confirm glioma diagnosis. Cancer-free controls were obtained through a contracting company by random-digit dialing using standard methods [25], and were frequency matched to cases on age (within 5 years), race/ethnicity, and sex. Eligibility criteria included the ability to speak English. Because frequency matching on sex was unsuccessful in the recruitment phase due to the higher incidence of glioma in males and a higher availability of cancer-free female controls, the study population was rematched on sex in the analysis phase. As a result, only a subset of available controls was used in our analyses.

Structured questionnaires were utilized to conduct detailed in-person or telephone interviews through which data on demographic factors, health characteristics, and familial attributes were obtained. About 20 mL of venous blood was collected from each participant (prior to chemotherapy or radiotherapy for cases). The original study was approved by the MD Anderson Cancer Center Institutional Review Board (IRB) and written informed consent was obtained from all participants. The analysis presented here was also approved by the Baylor College of Medicine IRB.

### Serology

Anti-HCMV IgG and IgM ELISA (enzyme-linked immunosorbent assay) kits (Alpco Diagnostics, Salem, New Hampshire) were used to determine anti-HCMV IgG levels and the presence or absence of anti-HCMV IgM (qualitative assay), according to manufacturers' instructions. Briefly, participants' samples were diluted at a ratio of 1:100 and added to antigen-coated wells in a microtiter plate. Bound antibodies were identified with peroxidase-conjugated rabbit anti-human IgG and TMB (3,3′,5,5′-tetramethylbenzidine) substrate. Absorbances were measured using a Tecan Infinite M200 plate reader at 450 nm. These assays were repeated for a random sample of participants, and the results had high test–retest reliability (*r* > 0.9). Serologic analyses were conducted without identification of case–control status.

### Statistical analysis

Differences in the distributions of matching characteristics between cases and controls were tested using χ^2^ tests. Unconditional logistic regression models were utilized to estimate odds ratios and 95% confidence intervals (CIs) for the associations between glioma status and anti-HCMV IgG levels. First, regression models were run among all cases and controls to assess the effect of IgG and IgM positivity (yes/no). Then, individuals who were anti-HCMV IgG negative were dropped and models among IgG-positive individuals were run to evaluate the effects of three anti-HCMV IgG levels (<10, 10–29, ≥30 units/mL), overall and stratified by IgM positivity. These IgG categories were determined using the standard samples included in the enzyme-linked immunosorbent assay kit. Matching characteristics (age, sex, and race) were included in all multivariable models to control for residual confounding.

Survival analysis was also conducted to determine whether IgG levels and IgM positivity were associated with mortality risk among glioma cases. Kaplan–Meier survival curves were constructed to visualize survival probability over time, and log-rank tests were utilized to evaluate differences by IgG level and IgM status. Hazard ratios and 95% CIs were calculated using Cox proportional hazards regression, adjusting for age, race, and sex. We did not control for cancer-directed surgery, radiation, or chemotherapy, as these are unlikely to be associated with IgG or IgM levels at diagnosis, and therefore, would not be a data-based confounder. Log–log plots were used to test the proportional hazards assumption. All *P*-values were two-sided with a 0.05 level of significance, and all statistical analyses were conducted using SAS version 9.1 (SAS Institute, Cary, NC).

## Results

Table 1 presents the distribution of matching characteristics among glioma cases (*n* = 362) and cancer-free controls (*n* = 462). Over half of all glioma cases had WHO grade IV tumors (53.9%, *n* = 195). Despite frequency matching, there were relatively small, although statistically significant, differences in the distribution of race/ethnicity, which we controlled for in the regression models. Neither anti-HCMV IgG nor IgM positivity was significantly associated with glioma risk (OR: 1.04, 95% CI: 0.78–1.39, and OR: 0.97, 95% CI: 0.72–1.31, respectively), adjusting for age, sex, and race/ethnicity. Among IgG-positive participants (*n* = 477; 207 cases, 270 controls), increasing anti-HCMV IgG levels were associated with decreasing glioma risk (*P* for trend = 0.0008), and those with the lowest level of anti-HCMV IgG (<10 U/mL) had the highest glioma risk, controlling for age, sex, and race/ethnicity (OR: 2.51, 95% CI: 1.42–4.43) (Table 2). These associations were also observed among IgM-positive individuals, but not among IgM-negative individuals. In a post hoc analysis in which the study population was restricted to cancer-free controls and WHO grade IV gliomas only, the ORs and trends observed were similar to those in Table 2.

**Table 1:** Population characteristics by glioma status

	Glioma cases (*n* = 362), *n* (%)	Controls (*n* = 462), *n* (%)	*P*^1^
Sex			
Male	204 (56.4)	228 (49.4)	0.05
Female	158 (43.7)	234 (50.7)	
Age			
<50 years	198 (54.7)	225 (48.7)	0.09
≥50 years	164 (45.3)	237 (51.3)	
Median (SD)	49 (13.8)	50 (13.1)	
Race			
Non-Hispanic white	305 (84.3)	413 (89.4)	0.03
Other	57 (15.8)	49 (10.6)	
Anti-HCMV immunoglobulin M			
Positive	232 (64.1)	300 (64.9)	0.80
Negative	130 (35.9)	162 (35.1)	
Anti-HCMV immunoglobulin G			
Positive	207 (57.2)	270 (58.4)	0.72
Negative	155 (42.8)	192 (41.6)	
WHO Glioma Grade			
II	97 (26.8)		
III	70 (19.3)		
IV	195 (53.9)		

HCMV, human cytomegalovirus.

1 Chi-squared test comparing cases to controls.

**Table 2:** Logistic regression models among anticytomegalovirus immunoglobulin G (IgG)-positive individuals, both overall and stratified by immunoglobulin M (IgM) positivity

	Logistic regression, adjusted odds ratios (95% CI)^1^		
	Overall	IgM positive	IgM negative
IgG level			
<10 U/mL	2.51 (1.42–4.43)^1^	3.73 (1.89–7.38)^2^	1.20 (0.41–3.54)
10–29 U/mL	1.51 (0.89–2.58)	1.40 (0.74–2.62)	1.64 (0.59–4.58)
≥30 U/mL	1.00 (reference)	1.00 (reference)	1.00 (reference)
Sex			
Female	1.00 (reference)	1.00 (reference)	1.00 (reference)
Male	1.05 (0.72–1.52)	1.20 (0.76–1.91)	0.86 (0.44–1.66)
Age			
<50 years	1.00 (reference)	1.00 (reference)	1.00 (reference)
≥50 years	0.81 (0.56–1.18)	0.81 (0.51–1.29)	0.84 (0.41–1.73)
Race			
Non-Hispanic White	1.00 (reference)	1.00 (reference)	1.00 (reference)
Other	1.29 (0.79–2.10)	1.18 (0.65–2.15)	1.31 (0.55–3.14)

1 Models compare antibody levels among anti-HCMV IgG-positive individuals only; all ORs are adjusted for age, sex, and race.

2 Significant *P* for trend (<0.05).

Approximately 72% of glioma cases died over the course of study follow-up, with a median survival time of about 14 months among those who died. Neither anti-HCMV IgG nor IgM positivity was significantly associated with mortality hazard over time (HR: 0.92, 95% CI: 0.70–1.22 and HR: 1.17, 95% CI: 0.89–1.55, respectively), controlling for age, sex, and race/ethnicity. Among IgG-positive glioma cases, anti-HCMV IgG levels were not predictive of survival over time, regardless of IgM status (Table 3). Kaplan–Meier curves (not shown) were consistent with model results. In a post hoc analysis in which the case population was restricted to WHO grade IV gliomas only, no significant associations were detected between mortality hazard over time and anti-HCMV IgG or IgM status or IgG levels.

**Table 3:** Cox proportional hazards regression models among anticytomegalovirus immunoglobulin G (IgG)-positive individuals, both overall and stratified by immunoglobulin M (IgM) positivity

	Cox proportional hazards regression, adjusted hazard ratios (95% CI)^1^		
	Overall	IgM positive	IgM negative
IgG level			
<10 U/mL	1.03 (0.56–1.88)	0.95 (0.47–1.90)	1.17 (0.34–4.01)
10–29 U/mL	0.94 (0.53–1.66)	0.67 (0.34–1.32)	1.82 (0.59–5.62)
≥30 U/mL	1.00 (reference)	1.00 (reference)	1.00 (reference)
Sex			
Female	1.00 (reference)	1.00 (reference)	1.00 (reference)
Male	1.29 (0.89–1.87)	1.24 (0.78–1.98)	1.23 (0.65–2.31)
Age			
<50 years	1.00 (reference)	1.00 (reference)	1.00 (reference)
≥50 years	2.88 (1.98–4.21)	3.28 (2.07–5.19)	2.53 (1.19–5.40)
Race			
Non-Hispanic White	1.00 (reference)	1.00 (reference)	1.00 (reference)
Other	0.64 (0.39–1.05)	0.75 (0.40–1.41)	0.49 (0.22–1.08)

1 Adjusted for age, race, and sex.

## Discussion

In this study, we found that anti-HCMV IgG levels were associated with glioma risk, especially among anti-HCMV IgM-positive individuals. We also detected a significant trend of increasing glioma risk with decreasing anti-HCMV IgG levels. However, we did not find similar associations for glioma survival. Nonetheless, our study contributes new evidence toward the potential associations between the direct and indirect impacts of HCMV infection and gliomagenesis.

To date, studies examining antibody response to HCMV in relation to glioma risk and survival have yielded relatively equivocal results [12, 20, 24]. Two previous analyses from the San Francisco Bay Area Adult Glioma Study (SFB) found inverse associations between anti-VZV IgG levels and glioma risk, but did not detect significant associations with anti-HCMV IgG levels [12,20]. A more recent study by Sjostrom et al. examined serology data from 197 adult glioma cases and 394 controls from Sweden and Denmark [26]. They also did not find a significant association between glioma or glioblastoma risk and anti-HCMV IgG levels. However, high anti-HCMV IgG levels have been found to be associated with longer survival among glioblastoma patients [27]. By contrast, our results indicated that lower levels of anti-HCMV IgG were associated with higher glioma risk, but not poorer survival, among IgG- and IgM-positive individuals. A primary difference between our study and the SFB studies is that they did not compare IgG-positive individuals with lower antibody levels to those with higher levels. Although they examined IgG status, they did not then exclude IgG-negative individuals from the IgG dose analyses. Given the ubiquity of HCMV infection [3], those who did not have a detectable IgG response may represent a different population compared with those who did with regard to length of time since initial HCMV infection, viral reactivation rates, viral load, or underlying immunological status (i.e., immunosuppressed, imbalanced Th1/Th2 response as among individuals with atopic conditions, etc.).

An additional difference between these previous studies and our own is that we also stratified our analyses on anti-HCMV IgM positivity. Among IgM-negative individuals, there was no significant association between IgG levels and glioma risk, and the overall association was largely explained by the IgM-positive stratum (Table 2). The presence of detectable levels of IgM may indicate a recent HCMV reactivation, as IgM is the first antibody generated after infection or reinfection [28]. Thus, it is possible that those who were IgM positive are individuals who are more likely to have HCMV reactivations throughout their lives, although this cannot be confirmed in this study. If viral reactivation is occurring, a stronger IgG response against the virus may be more important for actively preventing HMCV-related immunomodulation and other viral effects [24,27].

A key mechanism by which HCMV evades the immune system is through the production of proteins (coded by viral genes *TRL11/IRL11* and *UL119-UL118*) that share functional similarities with the host's Fc-gamma receptors (FCγRs) [24,27]. After the Fab domain of IgG binds to a viral or bacterial epitope, FCγRs on the surface of phagocytes interact with the Fc region of IgG in order to instigate phagocytosis and other effector responses. The functional similarities between HCMV's *TRL11/IRL11* and *UL119-UL118* protein products and FCγR may confer the virus the ability to competitively inhibit IgG binding to the host's FCγR and may thus reduce antibody-mediated effector immune functions. It follows that higher levels of IgG would likely allow for a greater probability of at least some of the antibodies binding with host FCγR, rather than the viral protein. As there are several hypotheses that may explain our observed associations [3, 23, 24, 27], more research into both the molecular mechanisms by which HCMV interferes with antibody-mediated immunity and host genetic susceptibility to HCMV infection and reactivation is necessary before we can understand the true importance of anti-HCMV IgG levels in gliomagenesis.

We acknowledge that our study has limitations. The most etiologically relevant time interval during which anti-HCMV IgG levels should be measured for studying glioma risk and survival is unknown. In our study (as in other studies on antibody levels and glioma risk), it is difficult to ascertain whether the lower IgG levels were present before the development of the tumor or whether these levels may be a product of the process of gliomagenesis. We are unable to determine whether our results may be explained by some kind of altered immune status associated with the disease, especially given that patients could have received steroids prior to diagnostic confirmation of glioma. This may have attenuated antibody levels among cases. However, as blood samples cannot feasibly be collected prior to diagnosis in a case–control study, there is little that can be done to mitigate this limitation. Another common limitation of studies that focus on the immune response to a specific virus is the unknown specificity of the antibody response toward the virus of interest. Potential cross-reactivity has been proposed as one possible explanation for observed associations between antibodies against certain viruses, such as VZV and HCMV, and glioma risk [20]. Additionally, it is possible that the relative timing (i.e., infancy, childhood, or adulthood) of initial infections with common viruses, such as HCMV, may be relevant to glioma etiology, potentially due to the subsequent effects on immune development. However, because several nonepidemiologic lines of evidence, including data from in vitro and animal studies, also support the hypothesis that HCMV infection and the related immunomodulatory sequelae may be involved in gliomagenesis, further research is needed. In particular, studies that have access to serial serology measurements over time are warranted because of the half-life of IgG and the possibility of differential HCMV reactivation rates between individuals.
